# The Burthen of Life

**Published:** 1890-07-12

**Authors:** 


### Words of Consolation.

### THE BURTHEN OF LIFE.

Are you ever so hard pressed by this life's struggles and trials that you pray for death? Life is very sweet to all, we know, but sometimes things weigh on us more heavily than at others, and then the prayer goes up, "It is enough now, O Lord; take away my life, for I am not better than my fathers."

We have two examples given us in the Bible of saints praying for death, but under very different circumstances. One was Simeon, whose eyes having beheld the new-born King, the Christ, the Saviour, casts them up to heaven with the joyous and well-nigh satisfied outburst, "Lord, now lettest Thou Thy servant depart in peace." He had received the blessing he had, all his life, been hoping for, and was now ready to yield up his spirit into the hands of Him who gave it. Few of us are able to enter into his feelings, much as we may admire his patient, beautiful character.

The other prayer for death came from Elijah, and was not wrung from him so much by one great trouble as by a number of small trials and discomforts heaped one on another, so that the prophet broke down utterly. His life was threatened by Queen Jezebel, but that was no new thing to him. Bodily wants and discomforts seem at this time to have overcome him. He had left his faithful servant Elisha behind him, and had been fleeing alone for his life all day, beneath a burning sun, across a dry and sandy desert. And now towards evening he cast himself down, weary, hungry, and thirsty, under a juniper tree. We cannot wonder that his spirit was cast down within him, or that he took a gloomy view of his spiritual progress, and felt that notwithstanding his recent triumphs over the prophets of Baal, and God's revelations to him, he was no better than his fathers, and therefore desired death.

But God was not angry with His desponding child, whose spiritual life seemed ebbing fast away. He gave His beloved one sleep, and placed bread and water by his pillow for his refreshment on awaking. His wants thus supplied by his Heavenly Father, Elijah rose up with fresh strength to do his Master's work.

We ask you, in the words of a modern writer, "Are you lying footsore and weary under your juniper tree, feeling as if God were dealing hardly with you? Do you find the daily cares, and worries, and perplexities of life almost more than you can bear? Take courage! Our God is a God that changeth not." He is our rest, and He is our refreshment always and for ever. As He gave rest to the weary prophet, so, if you bring your troubles to Him, you shall find rest for your soul. Your hungering heart will meet its satisfying portion in His love, and your thirsty spirit will drink of the fountain of life, and so be satisfied, and thus you also shall be strengthened, and go on your way rejoicing, willing to do the Master's work in the Master's way.

You may seem to have mis-spent all the moments of your day, which in the morning, at noontide, and at evening have been weary, though God shone over them all. The turmoil, the strife, the care, the burthens of life have to be borne, and well for us if we do not make them heavier by adding vain longings and vain fears to the scale. The Word shines through the gloom, and points to a resting place of hope and glory at Jesus' feet.

God never sends a sorrow
 Without the healing balm,
And bids us fight no battles
 But for the victor's palm.

But we by earth's mist blinded,
 Knew not His holy will,
Till o'er the troubled waters
 His voice said, "Peace, be still!"

